# Knowledge, preventive behavior and risk perception regarding COVID-19: a self-reported study on college students

**DOI:** 10.11604/pamj.supp.2020.35.2.23586

**Published:** 2020-06-11

**Authors:** Muhammed Elhadi, Ahmed Msherghi, Ahmed Alsoufi, Anis Buzreg, Ahmad Bouhuwaish, Ala Khaled, Abdulmueti Alhadi, Hind Alameen, Marwa Biala, Alsafa Elgherwi, Fatimah Elkhafeefi, Amna Elmabrouk, Abdulmuez Abdulmalik, Sarah Alhaddad, Ahmed Khaled, Moutaz Elgzairi

**Affiliations:** 1 Faculty of Medicine, University of Tripoli, University Road, Furnaj, 13275, Tripoli, Libya; 2 Faculty of Medicine, Tobruk University, Tobruk, Libya; 3 Faculty of Medicine, University of Zawia, Az-Zawia, Libya; 4 Faculty of Medicine, University of Benghazi, Benghazi, Libya; 5 Faculty of medicine, Libyan International Medical Graduate, Benghazi, Libya

**Keywords:** SARS-CoV-2, knowledge, public health, COVID-19, behavior, pandemic, awareness

## Abstract

**Introduction:**

There are a limited number of studies on the issues associated with the knowledge and self-practice preventive measures for COVID-19 among medical students. We aimed to determine the extent of knowledge, self-reported preventive behavior, and risk perception of the COVID-19 outbreak among college students in Libya.

**Methods:**

A cross-sectional study was conducted from April 20 to April 30, 2020. The participants were students of medical and non-medical subjects from Libyan educational institutes. Data on participants’ characteristics, knowledge, preventive behavior, and risk perception were collected.

**Results:**

Approximately 3669 participants completed the questionnaire, of which 2547 (69.4) were medical students and 1122 (30.6%) were non-medical students. The mean knowledge score on COVID-19 was 8.62 (SD: 1.26, range: 0-12), corresponding to 71.8% correct answers. A significant difference was observed between medical and non-medical students in terms of knowledge (p < 0.001). Overall, the knowledge score of the students differed significantly with respect to age, current year of study, and financial source (p < 0.05). The mean score of preventive behavioral measures toward COVID-19 (out of 8) was 7.42 (SD: 0.95, range: 0-8), and the overall preventive measure score was estimated to be approximately 7.42/8*100, which corresponds to 92.7% for both medical and non-medical students.

**Conclusion:**

Notably, college students were observed to have substantial knowledge, preventive behavior, and a positive attitude toward COVID-19. Government programs should aim to educate individuals from other sectors of the society to ensure the proper dissemination of knowledge on preventive safety measures, as this will help restrict and control the pandemic.

## Introduction

The severe acute respiratory syndrome coronavirus 2 (SARS-CoV-2) was first detected in Wuhan in the Hubei Province of China as the causative agent of coronavirus disease (COVID-19), a form of severe viral pneumonia, in December 2019 [1,2]. This was followed by rapid transmission worldwide, and soon the disease was declared a pandemic by the World Health Organization (WHO) in February 2020 [3]. On April 22, 2020, the WHO announced that 2.5 million cases with more than 160,00 deaths had been recorded [4]. The probability of transmission of COVID-19 differs based on the form and duration of exposure. Therefore, in areas where population dissemination is prevalent, preventive measures are necessary to reduce the chances of unintended exposure in the COVID-19 risk population [5-7]. Such steps are necessary to determine the cases of infection and for the rapid assessment and monitoring of COVID-19 transmission sources, and these could slow the spread of infection. These steps require the understanding of and precautions taken by the public to prevent the spread of the infection, and include measures such as social distancing, avoiding public gathering, and avoiding direct contact with suspected carriers [8,9]. In addition, several preventive measures are suggested to reduce the risk of transmission and the subsequent risk of disease, such as hand washing, avoiding touching the face, covering mouth while coughing, self-quarantine in cases of contact with individuals suspected or confirmed as COVID-19-positive, and disinfecting surfaces that may harbor the pathogen [10,11].

The development of vaccines against SARS-CoV-2 faces several financial, scientific, and ethical challenges, and may require several months of research and testing [12,13]. Therefore, preventive measures and risk perceptions are required to limit community transmission until vaccine and drug development processes reach a stage at which the prevention and control COVID-19 community transmission can be realized. A limited number of researchers have addressed issues such as the knowledge and self-practice preventive measures for COVID-19 among medical students [14]. Several studies have focused on healthcare workers rather than on communities and individuals [15,16], while certain studies have focused on community knowledge, attitude, and practice toward prevention of COVID-19 infection [17,18]. Students of medicine are expected to have substantial knowledge and awareness compared to the public as they are at a higher risk of contracting the virus owing to the nature of their work. Therefore, the risk of transmission should not be underestimated, particularly since healthcare workers could be a potential source of disease transmission in the community.

Medical students are the healthcare professionals of the future. Therefore, their knowledge and self-prevention practices should be assessed to ensure the effectiveness of preventive measures on COVID-19 outbreaks and community transmission. Moreover, compliance with preventive measures is a necessity and depends on the knowledge, preventive behavior, and risk perception of COVID-19 among individuals. There is a compelling need to understand the importance of general awareness about COVID-19 to promote COVID-19 control in countries and facilitate preventive measures. Major concerns have been raised with respect to the effects of these measures on controlling the spread of disease. This paper aims to determine the knowledge, self-reported preventive behaviors, and risk perception of the COVID-19 outbreak among medical students in Libya.

## Methods

**Study setting:** a cross-sectional survey was conducted between April 20 and April 30, 2020 with data submitted by 5000 medical and non-medical students using a Google form shared through email, mobile messages, and in print with students from 15 universities and colleges in Libya. The survey was conducted anonymously, and the participants were unaware of the study outcomes and provided consent after the study designed was explained. Questions on demographic data, including age, gender, type of study, year of study, availability of a stable financial source, the effect of civil war on COVID-19, and on a friend/family member with COVID-19 were included. Missing data or incomplete answers were excluded from the study. Additionally, the study participants were medical and non-medical students from the following disciplines; dentistry, pharmacy, medical technology, nursing, and veterinary sciences.

**COVID-19-related knowledge:** the extent of COVID-19-related knowledge was assessed in the first section through 12 items based on an earlier study on COVID-19 in China [18]. The questions were related to the knowledge of COVID-19, which consisted of two questions on the signs and symptoms, one on treatment, one on disease progression, three on disease transmission, three on prevention, and two on isolation care. Each correct answer was awarded 1 point, while incorrect answers or unanswered questions were awarded 0. The total knowledge score was 12. Reliability was calculated based on a Cronbach´s alpha value of 0.87.

**Preventive behavior and attitude:** approximately eight items from a previous survey were used to assess the preventive behavior [14]. Two questions were related to avoidance of public gathering, one on reducing the use of public transport, one on limiting shopping, one on cough etiquette, two on cleaning and hand hygiene practices, and one on the discussion of preventive measures with family members and acquaintances. Choosing the option “yes” added one point, while 0 was added for a “no” in each behavior-related question. The maximum prevention score was 8, with a score >6 indicating higher preventive behavior and appropriate awareness with respect to COVID-19. According to the method followed in a previous study, reliability was calculated based on Cronbach´s alpha value of 0.72. Two questions were added on the attitude toward COVID-19 control ability.

**Statistical analysis:** descriptive statistical measures, including frequency, percentage, and mean score, were used to report the findings. The chi-square test was used to determine the association between the categories. The independent-samples t-test was used to determine the difference between the means of two independent groups. A one-way ANOVA was calculated on participants’ score of knowledge and preventive behavior. Statistical analysis was performed using SPSS (IBM SPSS Statistics for Windows, Version 25.0; IBM Corp., Armonk, NY). P-value < 0.05 was considered statistically significant.

**Ethical consideration:** the ethical approval to conduct this study was obtained from the Bioethics Committee at the Biotechnology Research Center in Libya [Reference number: BEC-BTRC-141.4-8-2020]. All participants provided informed consent before participating in the study.

## Results

Out of the 5000 participants who received the questionnaire, approximately 3669 participants completed the questionnaire and provided completed data, with a response rate of 73.38% among the total study participants. Among those included, the mean age was 22.77 years (standard deviation (SD): 2.83, range: 17-30); out of 3669 included, 2923 (79.7%) were women, while 746 (20.3%) were male. The study participants included 2547 (69.4) medical students and 1122 (30.6%) non-medical students. The non-medical students were as follows: dentistry: 350 (8.3); pharmacy: 288 (7.8); medical technology: 271 (7.4); nursing: 250 (6.8); veterinary science: 8(0.2). The baseline characteristics are presented in Table 1. The mean score of knowledge on COVID-19 (out of 12) was 8.62 (SD: 1.26, range: 0-12), and the estimated overall knowledge was approximately 8.62/12 * 100, which corresponded to 71.8% correct answers. However, there was a significant difference between the answers provided by medical and non-medical students (p < 0.001). The mean ± SD score was 8.51 ± 1.15 for non-medical students and 8.67 ± 1.3 for medical students. In addition, for the answers to 9 of the 12 questions on knowledge, there was a significant difference between medical and non-medical students, where medical students provided correct answers at a higher rate. More than 94% of both categories of students were aware of the signs and symptoms of COVID-19. Additionally, there was a significant difference between the answers provided by medical and non-medical students to the question on disease progression; 95.1% of medical students were aware of the disease progression characteristics compared to 91.4% non-medical students. More than 97% of individuals from both groups were aware of the necessity of public transport avoidance to reduce the risk of transmission. Approximately 97% of medical students and 98% of non-medical students were aware of the 14-day isolation process. Table 2 presents an overview of the knowledge questions and answer rate differences between the two groups. Overall, the knowledge score for the students differed significantly based on age, the current year of study, and status of financial source (p < 0.05). Among non-medical students, the score was not significantly different across the baseline factors. Among medical students, the age, current year of study, financial source, and COVID-19 patient among friends and/or family members differed significantly between the groups. Table 3 presents an overview of the differences in knowledge between the groups with respect to the characteristics.

**Table 1:** Characteristics of individuals in the study

Variable	Total	Non-Medical Students(%) n=1122		Medical Students(%) n=2547		p-value
**Age in mean ± SD**	22.77 ± 2.83	21.3 ± 2.21		23.42 ± 2.82		<0.001**
**Gender**		(n)	(%)	(n)	(%)	0.032*
			
Male	746	204	(18.2)	542	(21.3)	-
Female	2923	918	(81.8)	2005	(78.7)	-
**Current year of study:**						<0.001**
Preparatory year	250	156	(13.9)	94	(3.7)	-
Year1	472	203	(18.1)	269	(10.6)	-
Year2	491	231	(20.6)	260	(10.2)	-
Year3	582	236	(21)	346	(13.6)	-
Year4	749	206	(18.4)	543	(21.3)	-
Year5	828	63	(5.6)	765	(30)	-
Internship	297	27	(2.4)	270	(10.6)	-
**Constant financial source**						0.096
Yes	1359	438	(39)	921	(36.2)	-
No	2310	684	(61)	1626	(63.8)	-
**Will civil war complicate the COVID-19 situation?**						<0.001**
Yes	2705	784	(69.9)	1921	(75.4)	-
No	964	338	(30.1)	626	(24.6)	-
**Friend or/and family with COVID-19**						0.303
Yes	75	27	(2.4)	48	(1.9)	
No	3594	1095	(97.6)	2499	(98.1)	

**Table 2:** Questionnaire on knowledge toward COVID-19

Question	Non-Medical Students n=1122		Medical Students n=2547		p-value
**K1. The main clinical symptoms of COVID-19 are fever, fatigue, dry cough, and myalgia.**	(n)	(%)	(n)	(%)	0.008*
			
True	1057	(94.2)	2453	(96.3)	
False	49	(4.4)	63	(2.5)	
I don't know	16	(1.4)	31	(1.2)	
**K2. Unlike in common cold, stuffy nose, runny nose, and sneezing are less common in individuals infected with COVID-19.**					0.068
True	767	(68.4)	1800	(70.7	
False	261	(23.3)	509	(20)	
I don't know	94	(8.4)	238	(9.3)	
**K3. Currently, there is no effective cure for COVID-19, although early symptomatic and supportive treatment can help most patients recover from the infection.**					0.215
True	952	(84.8)	2215	(87)	
False	62	(5.5)	116	(4.6)	
I don't know	108	(9.3)	216	(8.5)	
**K4. Not all individuals with COVID-2019 will develop severe infection. Only the elderly, or individuals with chronic illnesses and/or obesity are more likely to contract severe infection**					<0.001**
True	1025	(91.4)	2412	(95.1)	
False	56	(5)	46	(1.8)	
I don't know	41	(3.7)	80	(3.1)	
**K5. Eating or contact with wild animals may result in the infection by SARS-CoV-2.**					0.051
True	394	(35.1)	810	(31.8)	
False	408	(26.4)	918	(36)	
I don't know	320	(28.5)	819	(32.2)	
**K6. Individuals with COVID-19 who do not have fever cannot infect other individuals.**					<0.001**
True	33	(2.9)	172	(6.8)	
False	1052	(93.8)	2251	(88.4)	
I don't know	37	(3.3)	124	(4.9)	
**K7. SARS-CoV-2 spreads via respiratory droplets of infected individuals.**					<0.001**
True	1043	(93)	2442	(95.9)	
False	54	(4.8)	32	(1.3)	
I don't know	25	(2.2)	73	(2.9)	
**K8. Residents can wear general medical masks to prevent infection by SARS-CoV-2.**					0.025*
True	105	(9.4)	1926	(75.6)	
False	919	(81.9)	405	(15.9)	
I don't know	98	(8.7)	216	(8.5)	
**K9. It is not necessary for children and young adults to take precautionary measures against COVID-19**					<0.001**
True	105	(9.4)	403	(15.8)	
False	909	(81.9)	1759	(69.1)	
I don't know	98	(8.7)	385	(15.1)	
**K10. To prevent contraction of COVID-19, individuals should avoid visiting crowded places, such as train stations, and avoid public transportation**					<0.001**
True	1110	(98.9)	2490	(97.8)	
False	8	(0.7)	7	(0.3)	
I don't know	4	(0.4)	50	(2)	
**K11. Isolation and treatment of people with COVID-19 are effective methods of reducing the spread of the virus**					<0.001**
True	1107	(98.7)	2484	(97.5)	
False	11	(1)	8	(0.3)	
I don't know	4	(0.8)	55	(2.2)	
**K12. People who have come in contact with an individual with COVID-19 should immediately isolate themselves in a proper place. In general, the observation period is of 14 days**					0.001*
Yes	1101	(98.1)	2470	(97)	
No	12	(1.1)	13	(0.5)	
I don't know	9	(0.8)	64	(2.5)	
**mean ± standard deviation (Total score)**	8.51 ± 1.15		8.67 ± 1.3		0.001*

**Table 3:** Differences in knowledge score according to study characteristics

		Total students n= 3669			Non-Medical Students n=1122			Medical Students n=2547		
Variable	Number of participants	Knowledge score (mean ± standard deviation)	t/F	p-value	Knowledge score (mean ± standard deviation)	t/F	p-value	Knowledge score (mean ± standard deviation)	t/F	p-value
**Age range**			23.08	<0.001**		0.857	0.355		15.64	<0.001**
< 23	1795 (48.9)	8.52 ± 1.14			8.49 ± 1.11			8.54 ± 1.15		
≥ 23	1874 (51.1)	8.72 ± 1.35			8.56 ± 1.23			8.75 ± 1.38		
**Gender**			0.532	0.466		1.054	0.305		0.164	0686
Male	746	8.59 ± 1.21			8.44 ± 1.26			8.65 ± 1.45		
Female	2923	8.59 ± 1.41			8.53 ± 1.12			8.67 ± 1.25		
**Current year of study:**			8.131	<0.001**		1.241	0.282		6.08	0686
Preparatory year	250	8.37 ± 1.35			8.44 ± 1.1			8.26 ± 1.69		
Year 1	472	8.54 ± 1.17			8.53 ± 1.15			8.55 ± 1.19		
Year 2	491	8.47 ± 1.17			8.38 ± 1.22			8.55 ± 1.11		
Year 3	582	8.61 ± 1.21			8.57 ± 1.17			8.63 ± 1.24		
Year 4	749	8.65 ± 1.10			8.53 ± 1.06			8.69 ± 1.11		
Year 5	828	8.67 ± 1.13			8.76 ± 1.21			8.67 ± 1.13		
Internship	297	9.01 ± 1.98			8.66 ± 0.96			9.04 ± 2.02		
**Stable financial source**			17.101	<0.001**		0.284	0.594		27.772	<0.001**
Yes	1359	8.55 ± 1.24			8.53 ± 1.18			8.85 ± 1.35		
No	2310	8.73 ± 1.28			8.49 ± 1.09			8.56 ± 1.26		
**Friend or/and family member with COVID-19**			2.251	0.134		0.025	0.875		3.954	<0.001**
Yes	75	8.84 ± 2.06			8.48 ± 1.55			8.66 ± 1.27		
No	3594	8.61 ± 1.23			8.51 ± 1.14			9.04 ± 2.28		

The mean score of preventive behavioral measures toward COVID-19 (out of 8) was 7.42 (SD: 0.95, range: 0-8), and the overall preventive measure score was estimated to be approximately 7.42/8*100, which corresponds to 92.7% for both medical and non-medical students. Table 4 presents an overview of the questions on preventive measures and the answer rate differences between the two groups. However, there was a statistically significant difference between the two groups with respect to two questions. Approximately 97% individuals from both groups reported reducing the use of public transport and shopping activities. Approximately 96% of individuals from both groups reported avoiding coughing around other individuals and practicing cough etiquette. More than 98% of them reported avoidance of crowded places or places with mass gatherings. In addition, approximately 87% non-medical students and 84% medical students reported an increase in the use of cleaning and disinfecting items. In addition, more than 91% of all students reported washing their hands more frequently than usual. In addition, more than 88% reported engaging in discussions on COVID-19 prevention with their friends and family members.

**Table 4:** Questionnaire on preventive behavior and attitude toward COVID-19

Question	Non-Medical Students n=1122		Medical Students n=2547		p-value
**P1. I cancelled or postponed meetings with friends, eating-out, and sports events**	(n)	(%)	(n)	(%)	0.009*
			
Yes	942	(84)	2221	(87.2)	
No	180	(16)	326)	(12.8)	
**P2. I reduced the use of public transportation.**					0.999
Yes	1096	(97.7)	2488	(97.7)	
No	26	(2.3)	59	(2.3)	
**P3. I went shopping less frequently.**					0.083
Yes	1087	(96.9)	2492	(97.8)	
No	35	(3.1)	55	(2.2)	
**P4. I avoided coughing in public places as much as possible.**					0.706
Yes	1085	(96.7)	2469	(96.9)	
No	37	(3.3)	78	(3.1)	
**P5. I avoided places with mass gatherings.**					0.402
Yes	1106	(98.6)	2519	(98.9)	
No	16	(1.4)	28	(1.1)	
**P6. I increased the frequency of cleaning and disinfecting items that are frequently touched with hands (i.e. door handles and surfaces).**					0.034*
Yes	979	(87.3)	2154	(84.6)	
No	143	(12.7)	393	(15.4)	
**P7. I washed hands more often than usual.**					0.856
Yes	1028	(91.6)	2329	(91.4)	
No	94	(8.4)	218	(8.6)	
**P8. I discussed COVID-19 prevention measures with my family members and friends.**					0.231
Yes	988	(88.1)	2277	(89.4)	
No	134	(11.9)	270	(10.6)	
**Total score (mean ± standard deviation)**	7.40 ± 0.93		7.43 ± 0.96		0.343
**A1. Do you agree that eventually, COVID-19 control will be successful?**	(n)	(%)	(n)	(%)	0.019
			
Yes	984	(87.7)	2159	(84.8)	
No	138	(12.3)	388	(15.2)	
**A2. Are you confident that Libya can win the battle against COVID-19?**					0.485
Yes	837	(74.6)	1872	(73.5)	
No	285	(25.4)	675	(26.5)	

Table 5 outlines the difference in major characteristics according to the difference in the mean scores from the preventive measure questionnaire. There was a significant difference with respect to gender and age (p < 0.05) in the overall group. Among the non-medical students, the mean difference between the categories was statistically significant with respect to gender, financial source, and having a COVID-19-infected family member and/or friend. Among the medical students, there was a statistically significant difference with respect to age, gender, and year of study. Table 5 presents an overview of the differences in preventive behaviors based on the study characteristics. Approximately 84% of the non-medical students and 87.2% of the medical students reported canceling or postponing their meetings and outdoor activities as a preventive measure. The majority of participants agreed with the statement that COVID-19 could be controlled (87.7% of non-medical students and 84.8% of medical students). Approximately 74.6% of non-medical students and 73.5% of medical students agreed with the statement that the COVID-19 outbreak in Libya could be controlled. However, there was a significant association of the attitude score with both genders and with the financial status (P < 0.05). The categories of age, and having a friend/family member with COVID-19 had no significant association with the attitude score (p > 0.05).

**Table 5:** Differences in preventive score according to study characteristics

		Total students n= 3669			Non-Medical Students n=1122			Medical Students n=2547		
Variable	Number of participants	Preventive behavior score (mean ± standard deviation)	t/F	p-value	Preventive behavior score (mean ± standard deviation)	t/F	p-value	Preventive behavior score (mean ± standard deviation)	t/F	p-value
**Age range**			9.41	0.002*		0.004	0.948		11.21	0.001*
< 23	1795 (48.9)	7.38 ± 0.99			7.40 ± 0.89			7.35 ± 1.06		
≥ 23	1874 (51.1)	7.47 ± 0.91			7.41 ± 1.01			7.49 ± 0.88		
**Gender**			59.933	<0.001**		14.172	<0.001**		46.374	<0.001**
Male	746	7.19 ± 1.18			7.18 ± 1.19			7.19 ± 1.18		
Female	2923	7.49 ± 0.87			7.45 ± 0.85			7.51 ± 0.88		
**Current year of study**			2.01	0.061		1.354	0.23		2.49	0.021*
Preparatory year	250	7.3 ± 1.11			7.39 ± 0.75			7.13 ± 1.51		
Year 1	472	7.37 ± 0.97			7.38 ± 0.95			7.37 ± 0.99		
Year 2	491	7.41 ± 0.93			7.35 ± 0.98			7.46 ± 0.89		
Year 3	582	7.38 ± 0.97			7.38 ± 0.96			7.39 ± 0.99		
Year 4	749	7.47 ± 0.91			7.54 ± 0.82			7.44 ± 0.93		
Year 5	828	7.48 ± 0.88			7.25 ± 1.23			7.5 ± 0.85		
Internship	297	7.47 ± 1.02			7.55 ± .84			7.46 ± 1.04		
**Stable financial source**			3.73	0.054		11.57	0.001*		0.017	0.898
Yes	1359	7.46 ± 0.85			7.52 ± 0.76			7.44 ± 0.89		
No	2310	7.40 ± 1.01			7.33 ± 1.01			7.43 ± 1.00		
**Friend or/and family member with COVID-19**			1.88	0.169		7.41	0.007*		0.082	0.775
Yes	75	7.28 ± 1.14			6.92 ± 1.46			7.43 ± 0.96		
No	3594	7.43 ± 0.94			7.41 ± 0.91			7.47 ± 0.87		

## Discussion

To the best of our knowledge, this is the first study conducted in Africa that discusses knowledge, self-preventive measures, and attitudes toward COVID-19 among college students, who constitute an integral part of an educated society. Overall, 71.8% of the study participants were aware of COVID-19, while more than 92.7% of both medical and non-medical students were taking preventive measures against COVID-19 to reduce the risk of infection transmission. Our study provides an overview of the knowledge of medical students, who will work as physicians in future, and are currently engaged in hospitals and at a high risk of infection transmission; the study compared their knowledge, preventive measures, and attitude toward COVID-19 to that of non-medical students to determine the difference. A majority of the participants (87.7% of non-medical students and 84.8% of medical students) opined that the COVID-19 pandemic could be controlled successfully, while a lower percentage of participants (74.6% non-medical students and 73.5% medical students) believed that the disease could be controlled in Libya. Contrastingly, the preventive behavior score among students was encouraging, as indicated by a high mean score of 7.4 ± 0.93 in non-medical students and 7.43 ± 0.96 of medical student out of 8, which corresponds to approximately 7.4/8 *100 = 92.5% of non-medical student and 7.43/8 *100 = 92.87% of medical students; this suggests that the students have a positive preventive behavior and attitude toward COVID-19. Students in their internship year had better knowledge and greater preventive behavior owing to the extent of time they spend at the medical department, where they work at a higher risk of exposure and encounter more cases. Therefore, their knowledge and attitude are attributed to their experience.

Approximately 97% participants from both groups reported avoidance of crowds and shopping, which displays the high levels of caution among the individuals. In addition, we identified several factors that can be associated with the preventive behavior and knowledge of the participants toward COVID-19. Our findings are necessary to understand the current state of public health awareness and to determine the need for proper dissemination of knowledge and awareness among students who are medical practitioners of the future and may possibly come in contact with infected individuals during their rotation in the hospitals. As medical students are at a higher risk of contracting infection and disease transmission owing to the course of study in hospitals, their knowledge and awareness of preventive measures are crucial for reducing the risk of community spread of SARS-CoV-2. Despite the healthcare situation and civil war in Libya, college students achieved high scores in knowledge, preventive behavior, and attitude toward COVID-19 infection. There was no significant difference between the rates of correct answers to most questions asked to either medical or non-medical students, which displays the substantial overall knowledge among college students. Their level of knowledge, preventive behavior, and attitude owing to their active learning and sources of information on COVID-19 yielded positive results. A similar study conducted on a Chinese population reported an overall knowledge of 90% [18]. Another unpublished study conducted by Haque et al. on a Bangladeshi population reported an overall knowledge score of 54.87% toward COVID-19 [19]. In addition, another study conducted on the Nepalese population reported an overall knowledge score ranging from 60.0-98.7% and attitude scores ranging from 77.9-96.4%. The study also confirmed that participants with a medical degree had greater knowledge than non-medical participants [20]. Another study conducted on an Egyptian population comprising 559 participants reported the mean and standard deviation of knowledge score as 16.39±2.63, ranging from 7 to 22, which corresponds to approximately 74.5% overall knowledge among participants regarding COVID-19 [17]. Another study in Thailand reported 67.9% as the overall level of knowledge toward COVID-19 prevention and only 29.5% were reported to have substantial knowledge about the preventive measures for COVID-19, which is lower than that in our study findings [21]. Another study conducted among Iranian medical students showed that 79.6% of medical students had adequate knowledge about COVID-19 [14].

Our study reported high scores of attitude and preventive behavior toward COVID-19, which corresponds to a lower potential risk rate for COVID-19. In addition, more than 88% of the study participants reported discussing the preventive measures with their families and friends, which can effectively increase awareness about the current situation. This indicates the importance of health education that could improve the prevention behavior toward COVID-19 in society. The health education approach is a common method adopted for reducing the risk of transmission of infectious diseases. The study had a large sample size and involved surveying students from approximately 15 colleges and universities during the COVID-19 outbreak in Libya. In addition, our sample was representative of those from major cities, and the study yielded a generalized result. However, our sample predominantly comprised female students, 2923 (79.7%) overall, between the two groups. In addition, we included only college students who are likely to have better knowledge and information about COVID-19. Therefore, this may have limited our estimation of the knowledge of people who do not have access to a college education or the general population. Additionally, the comparison between medical and non-medical students did not display significant differences for most questions despite the fact that medical students were more likely to have greater knowledge owing to the nature of their profession and academic pursuit; this could be attributed to the larger sample size of medical students, where 69.4% of the sample size comprised medical students and 30.6% were non-medical students. However, our study covered populations from major cities and universities and can be considered as a generalized representation of the Libyan population irrespective of residence state.

## Conclusion

Our study revealed that college students have substantial knowledge, preventive behavior awareness, and a positive attitude toward COVID-19. A majority of them also expressed their optimism regarding the control of COVID-19. In addition, there was a significant difference between the scores of the medical and non-medical student groups; however, the majority of correct answers did not differ significantly. Government programs should aim to educate individuals from other sectors of the society and the general population to ensure that preventive safety measures are adopted, as this will help in controlling the pandemic.

### What is known about this topic

- The extent of community knowledge, attitude, and preventive behavior in COVID-19 are major concerns;
- Several studies have reported knowledge, attitude, and preventive behavior among individuals without specific emphasis on college students.

### What this study adds

- Our study revealed that college students have substantial knowledge, preventive behavior awareness, and a positive attitude toward COVID-19;
- There was a significant difference between the knowledge, preventive behavior, and positive attitude scores between medical and non-medical student groups.

## Competing interests

The authors declare no competing interests.
